# Interictal EEG features as computational biomarkers of West syndrome

**DOI:** 10.3389/fped.2024.1406772

**Published:** 2024-06-05

**Authors:** Jiaqing Li, An-an Ping, Yalan Zhou, Tangfeng Su, Xiaoli Li, Sanqing Xu

**Affiliations:** ^1^ Department of Pediatrics, Tongji Hospital, Tongji Medical College, Huazhong University of Science and Technology, Wuhan, China; ^2^ State Key Laboratory of Cognitive Neuroscience and Learning & IDG/McGovern Institute for Brain Research, Beijing Normal University, Beijing, China

**Keywords:** hypsarrhythmia, interictal EEG, West syndrome, power spectrum, cross-frequency coupling, entropy

## Abstract

**Background:**

West syndrome (WS) is a devastating epileptic encephalopathy with onset in infancy and early childhood. It is characterized by clustered epileptic spasms, developmental arrest, and interictal hypsarrhythmia on electroencephalogram (EEG). Hypsarrhythmia is considered the hallmark of WS, but its visual assessment is challenging due to its wide variability and lack of a quantifiable definition. This study aims to analyze the EEG patterns in WS and identify computational diagnostic biomarkers of the disease.

**Method:**

Linear and non-linear features derived from EEG recordings of 31 WS patients and 20 age-matched controls were compared. Subsequently, the correlation of the identified features with structural and genetic abnormalities was investigated.

**Results:**

WS patients showed significantly elevated alpha-band activity (0.2516 vs. 0.1914, *p *< 0.001) and decreased delta-band activity (0.5117 vs. 0.5479, *p *< 0.001), particularly in the occipital region, as well as globally strengthened theta-band activity (0.2145 vs. 0.1655, *p *< 0.001) in power spectrum analysis. Moreover, wavelet-bicoherence analysis revealed significantly attenuated cross-frequency coupling in WS patients. Additionally, bi-channel coherence analysis indicated minor connectivity alterations in WS patients. Among the four non-linear characteristics of the EEG data (i.e., approximate entropy, sample entropy, permutation entropy, and wavelet entropy), permutation entropy showed the most prominent global reduction in the EEG of WS patients compared to controls (1.4411 vs. 1.5544, *p *< 0.001). Multivariate regression results suggested that genetic etiologies could influence the EEG profiles of WS, whereas structural factors could not.

**Significance:**

A combined global strengthening of theta activity and global reduction of permutation entropy can serve as computational EEG biomarkers for WS. Implementing these biomarkers in clinical practice may expedite diagnosis and treatment in WS, thereby improving long-term outcomes.

## 1 Introduction

West syndrome (WS), is a severe developmental and epileptic encephalopathy that occurs in infancy or early childhood, affecting 2–5/10,000 newborns (1). It is characterized by clustered epileptic spasms, interictal hypsarrhythmia on scalp electroencephalogram (EEG), and neurodevelopmental arrest or psychomotor delay (2). WS is drug-resistant with only a third of patients experiencing seizure remission after treatement. Cognitive impairments persist, and fewer than 20% of patients can attend school normally, while approximately 20% of patients develop autism spectrum disorder (3). At least 20% of WS patients do not survive to adulthood (4). Early diagnosis and appropriate intervention are crucial for achieving a positive outcome for WS patients.

Hypsarrhythmia, as first introduced by Gibbs and Gibbs (5), is regarded as the EEG hallmark of WS. It is characterized by disorganized high-amplitude slow waves and multi-focal spikes without interhemispheric synchronization. Many variants of hypsarrhythmia, including typical hypsarrhythmia and modified hypsarrhythmia, have been described (6). Despite its common use, the term “hypsarrhythmia” lacks quantifiable definitions, therefore, clinicians rely on visual interpretation of electrographic findings for the diagnosis of WS. Yet, due to the wide variability in temporal and spatial patterns, the interrater reliability is remarkedly low (7, 8).

Advances in digital EEG recording and signal processing techniques have enabled in-depth investigation of brain signals. The derivation of a variety of quantifiable features not only enhances our understanding of neurophysiology in health and diseases (9) but also provides alternative or supplementary biomarkers for clinical evaluations (10). For instance, recent studies revealed that WS patients exhibit stronger delta/alpha activity (11) and enhanced high-frequency oscillation (12, 13). It has been also proposed that a stronger coupling between high-frequency oscillation with slow wave activity could serve as a predictor of drug responsiveness in WS patients (14). Additionally, WS patients demonstrate abnormal functional connectivity (15), and reduced EEG complexity, i.e., lower entropy (10, 16). However, further studies are necessary to validate whether these observations could be repeated in larger cohorts and whether they could be used for differential diagnosis in clinical practices.

The etiology of WS is diverse and can be attributed to genetic, structural, infectious, metabolic, and immunologic abnormalities. Accurate etiologic determination is crucial for selecting the appropriate intervention strategy and achieving positive outcomes. However, in approximately one-third of WS patients, the etiological cause remains unknown even after extensive investigation (17). Furthermore, different factors may act in association and cannot be reliably distinguished. It is also unclear whether different etiological causes could result in distinctive EEG patterns in WS.

In this study, we aimed to investigate whether there are specific interictal EEG biomarkers of WS independent of hypsarrhythmia and whether these EEG patterns could be attributed to structural or genetic abnormalities. To this end, we analyzed EEG recordings from 31 WS patients with structural brain aberrancies and/or genetic abnormalities and compared them to 20 age-matched controls. Our focus was on linear features including power spectrum, cross-frequency coupling, bi-channel coherences as well as non-linear characteristics: the entropy of EEG data. Subsequently, we explored the correlation of these features with structural and genetic abnormalities.

## 2 Materials and methods

### 2.1 Study population

This study was conducted at the Department of Pediatric Neurology in Tongji Hospital, affiliated to Tongji Medical College, a tertiary medicine center in China. We retrospectively reviewed the clinical records of 123 children diagnosed with WS at our center between January 2021 and August 2023. All children underwent both magnetic resonance imaging (MRI) and genetic testing. From this group, 87 patients with identified structural brain abnormalities and/or genetic findings were selected for further evaluation. Two independent, board-certified clinical neurophysiologists re-evaluated the video-EEG recordings of these patients. Ultimately, 31 patients with discontinous hypsarrhythmia patterns on interictal EEG, confirmed by both neurophysiologists, were enrolled in the final analysis of this study. The median age (interquartile range) of the WS patients was 8 (6, 10) months, with 15 (48%) of them being female. Nonspecific MRI abnormalities were observed in 13 (42%) patients, and causative structural abnormalities associated with WS were observed in 9 (29%) patients. Genetic alterations associated with WS were identified in 17 (55%) patients through whole-exome sequencing (MyGenostics Co., Ltd). The clinical characteristics of the group of patients are summarized in Table 1.

**Table 1:** Clinical features of WS patient group.

NO.	Gender	Age (month)	Brain MRI abnormalities	Genetic mutation
1	Female	10	Negative	*MED13l*
2	Female	5	Negative	*GABRG2*
3	Male	6	Negative	*KCNQ2*
4	Male	10	Negative	*GRIN2D*
5	Female	9	Negative	*ALG11*
6	Male	5	Negative	*TSC2*
7	Male	8	Negative	*CACNA1A*
8	Male	15	Negative	*GRIN2A*
9	Female	8	Negative	*STXBP1*
10	Female	20	Right hippocampus sclerosis	*IRF2BPL*
11	Male	9	Brain atrophy	*CAMK2A*
12	Female	10	White matter volume loss	*ATN1*
13	Male	6	Slight ventriculomegaly	*GRIN1*
14	Male	7	Delayed myelination, bilateral frontal-parietal encephalomalacia	*TSC2*
15	Male	8	Abnormal occipital signal^a^	*CDKL5*
16	Female	12	Mild ventricular dilation	*SCN1A*
17	Female	7	Subependymal multiple nodules^a^	*TSC2*
18	Male	8	Choroid plexus cysts	Negative
19	Male	20	Signal abnormality in the left hippocampus	Negative
20	Female	13	Periventricular leukomalacia^a^	Negative
21	Female	6	Brain atrophy, signal abnormality in the bilateral basal ganglia^a^	Negative
22	Male	6	Pachygyria	Negative
23	Female	3	Slight ventriculomegaly^a^	Negative
24	Male	21	Porencephalic cyst^a^	Negative
25	Male	6	Delayed myelination	Negative
26	Female	4	Lissencephaly	Negative
27	Male	3	Increased extracerebral space, delayed myelination^a^	Negative
28	Female	15	Right hippocampus sclerosis	Negative
29	Female	10	Brain atropy	Negative
30	Male	7	Focal cortical dysplasia^a^	Negative
31	Female	5	Bilateral subcortical heterotopia^a^	Negative

^a^ Causative structural abnormalities in our WS patient cohort.

20 healthy subjects were enrolled as the control group. The subjects were physically and mentally healthy with no symptoms or history of any neurological disorders. The EEG examination was normal. The median age (interquartile range) was 7 (4, 11) months, with 10 (50%) of the subjects being female.

This study was approved by the Ethics Committee in Tongji Hospital of Tongji Medical College, Huazhong University of Science and Technology. Written informed consent was obtained from the statutory guardian of each participant.

### 2.2 EEG recording and preprocessing

EEG signals were recorded using the NicoletOne EEG system, which utilized nineteen scalp EEG electrodes (Fp1, Fp2, F7, F8, F3, F4, C3, C4, T3, T4, P3, P4, T5, T6, O1, O2, Fz, Cz, and Pz) following the international 10–20 electrode placement system. The data were recorded with a sampling rate of 250 Hz and band-pass filtering between 0.5–70 Hz, along with a notch filter at 50 Hz. For each participant in the control group, a continuous resting-state EEG recording of at least 30 min was obtained. 5 epochs of 3 min artifacts-free EEG recordings from each subject were analyzed. For each subject in the WS group, video EEG recording of at least 4 h in duration was obtained. And after meticulously reviewing, EEG segments during wakefulness that did not display obvious hypsarrhythmia or hypsarrhythmia variants was selected for downstream analysis (Supplementary Figure S1). On average, 37% (±17%) of interictal EEG epochs from these patients was free of hypsarrhythmia or hypsarrhythmia variants.

EEG data were processed using scripts based on the EEGLAB toolbox (18) in MATLAB (Mathworks Inc., USA, version 2021b). Briefly, the EEG signals were initially transformed to a common average reference and subsequently filtered to 1–45 Hz with a Hamming windowed finite impulse response (FIR) filter provided in EEGLAB.

### 2.3 Data analysis

#### 2.3.1 Power spectrum analysis

Power spectrum analysis was performed using Welch's power spectral density estimate method (PSD) in a 2-s Hamming window with 25% overlap. We calculated the sum of powers on the following frequency bands: Delta, 1 Hz ≤ Fq < 4 Hz; Theta, 4 Hz ≤ Fq < 8 Hz; Alpha, 8 Hz ≤ Fq < 13 Hz; Beta, 13 Hz ≤ Fq < 30 Hz. The relative power (Equation 1) of one frequency band was defined as the sum of power within that band divided by the sum of power in the whole band:

(1) $$RP(\;f_{1},f_{2})=\frac{P(\;f_{1},f_{2})}{P(1,45)}$$

where *p* (·) indicates the power, RP (·) indicates the relative power and $f_{1}$, $f_{2}$ indicate the low and high frequency, respectively. Relative power values across all 2-s epochs were then averaged to improve robustness.

#### 2.3.2 Cross-frequency coupling

Cross-frequency coupling was assessed using wavelet-bicoherence as reported previously (19). To analyze the signals, we divided them into 2-s epochs with a 75% overlap. Within each epoch, we computed bicoherence values (Equation 2) for frequency pairs ranging from 1 Hz–45 Hz, with a step of 1 Hz and a bandwidth of 2 Hz. For every 3 min EEG segment, we obtained a wavelet bicoherence matrix, denoted as “*b*”, where each element $b(f_{p},f_{q})$ represents the bicoherence value at the bifrequency pair $\left(f_{p},f_{q}\right)$. Finally, the filtered wavelet bicoherence value (FIWBIC) is defined as:

(2) $$b=\sum \sum b_{xxx\;\;}^{2}(\;f_{p},f_{q})$$

We calculated FIWBIC from six pairs of frequency bands: delta-alpha, delta-beta, delta-theta, theta-alpha, theta-beta, and alpha-beta. FIWBIC values across 2-s epochs were averaged for robustness.

#### 2.3.3 Bi-channel coherence

Estimation of bi-channel coherence (Equation 3) was performed using the magnitude-squared coherence method in Matlab (mscohere) on delta/theta, alpha, and beta bands. Briefly, the signals were divided into a series of 2-s epochs. For $P_{x}(f)$ and $P_{y}(f)$ as power spectral densities of two channels at certain band, mscohere is defined as:

(3) $$C_{xy}(f)=\frac{{\left|P_{xy}(f)\right|}^{2}}{P_{x}(f)P_{y}(f)}$$

We calculated bi-channel coherence from three frequency bands: delta-theta, alpha, and beta. Bi-channel coherence values across 2-s epochs were then averaged for robustness.

#### 2.3.4 Entropy analysis

In this study, four entropy features, that is, approximate entropy (ApEn), sample entropy (SaEn), permutation entropy (PeEn), and wavelet entropy (WaEn) were calculated. Detailed calculation procedures can be found in (20). Briefly, ApEn was computed by embedding the signal into the phase space and calculating the rate of increment in the phase space patterns within a predefined value *r* as the embedding dimension of phase space increases from *m* to *m* + 1. SaEn was based on ApEn but modified to improve robustness by eliminating self-matches. PeEn quantifies the randomness by dividing a given time series into a train of ordinal patterns to describe the order relations between the present and a fixed number of equidistant past values. WaEn is calculated by first decomposing the signal using wavelet transformation, and then computing the Shannon entropy of the wavelet coefficients, providing a time-frequency representation of a dynamic process. Complexity values calculated from all 2-s epochs (separately per entropy type) were averaged to enhance robustness.

### 2.4 Statistics

**Figure 1:**
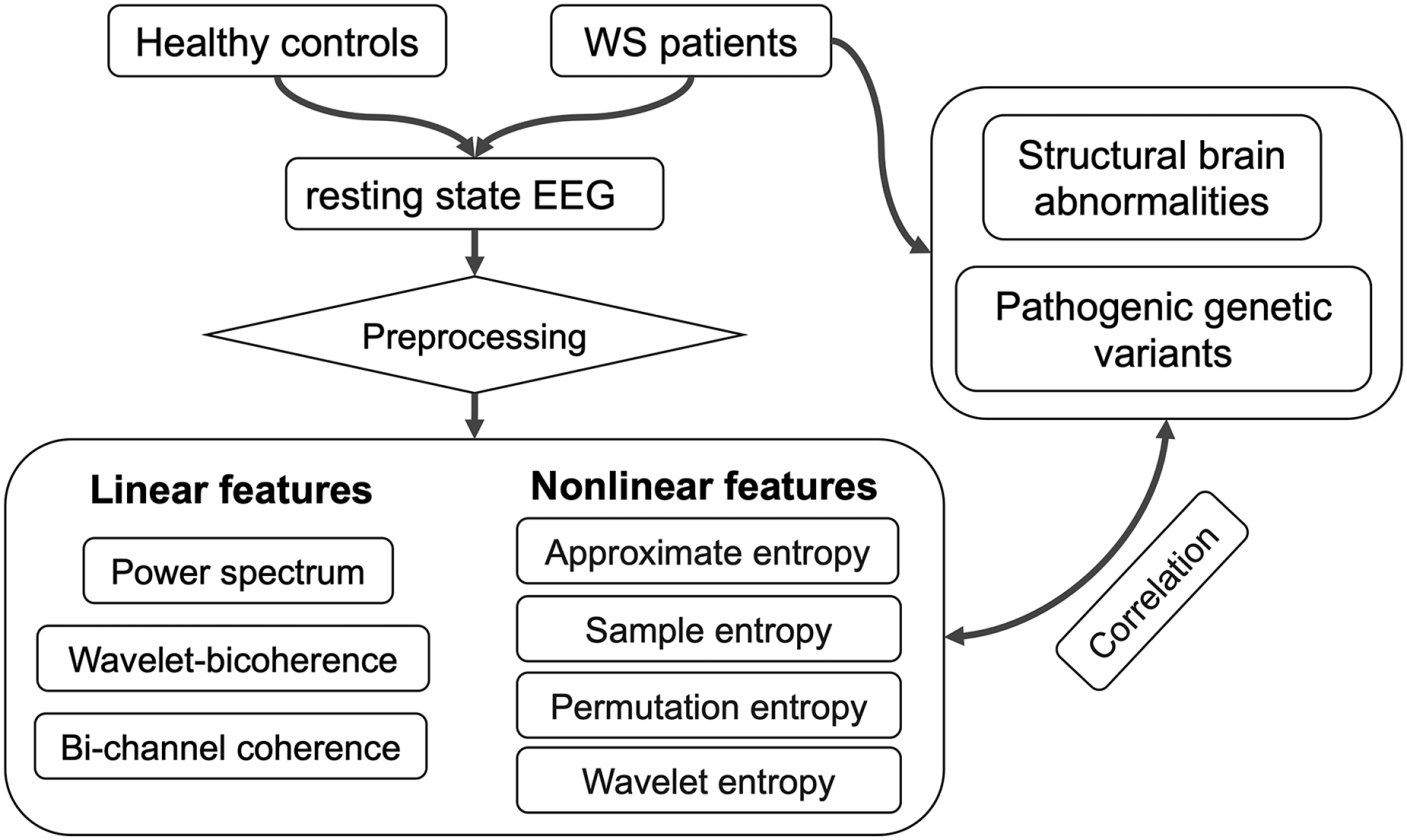
Data processing pipeline of the present study.

Statistical analysis was performed using MATLAB. Specifically, for each EEG feature, a two-sample *t*-test was utilized to assess the difference between patients and the control group, followed by a False Discovery Rate (FDR) correction using the Benjamini-Hochberg procedure (mafdr). To evaluate the association between EEG features and structural-genetic etiologies, multivariate linear regression was employed, and FDR was corrected as mentioned above. A schematic illustration of the data processing pipeline in the present study is depicted in Figure 1.

## 3 Results

### 3.1 Interictal EEG of WS patients revealed altered power spectrum, decreased cross-frequency coupling and abnormal brain connectivity

**Figure 2:**
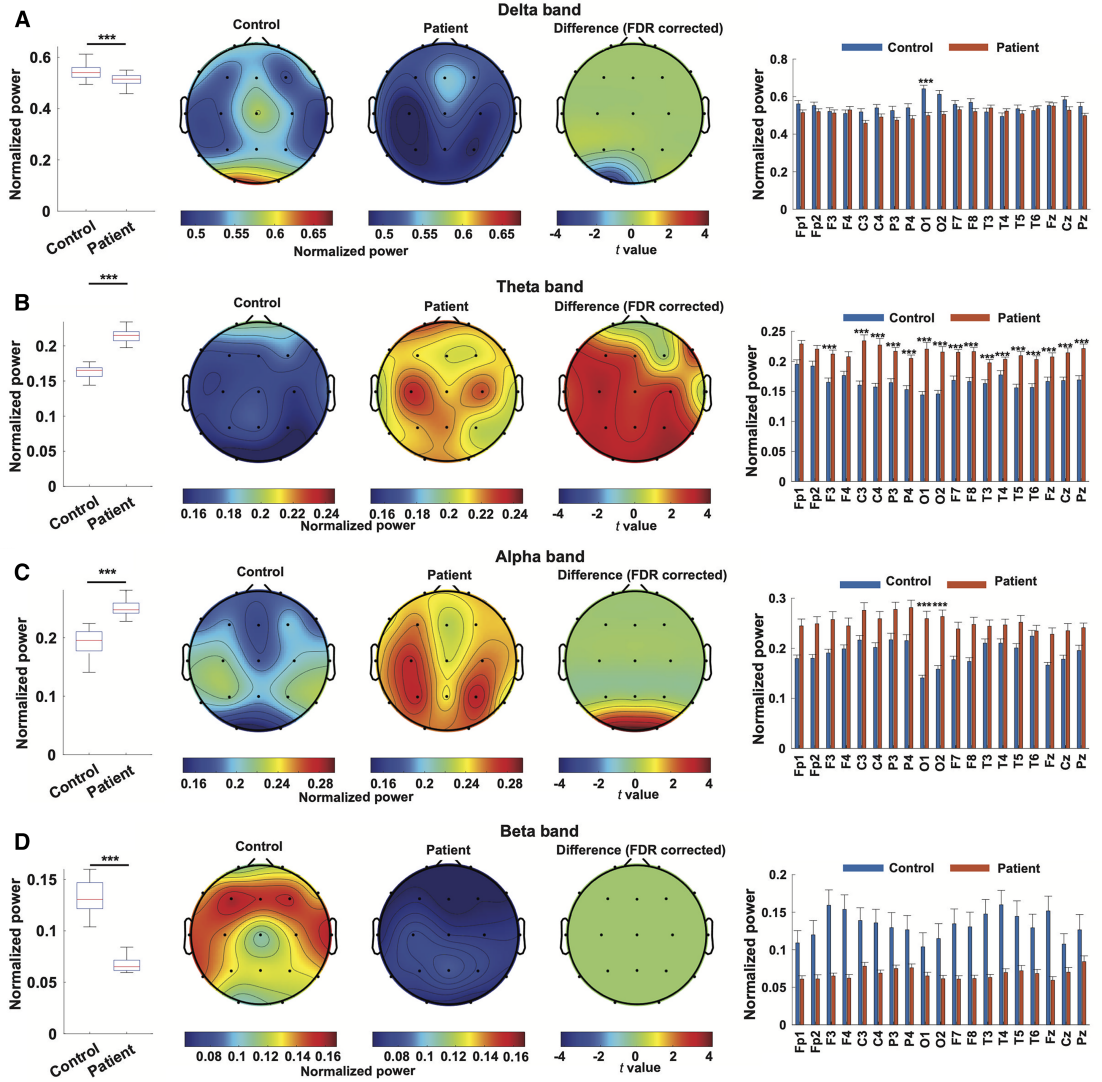
Power spectrum characterization of EEG from WS patients. Normalized power in the delta band (**A**), theta band (**B**), alpha band (**C**), and beta band (**D**) in EEG from WS patients and controls were compared. For every frequency band, a comparison of normalized power was depicted as a boxplot, followed by topographical maps of normalized power in the specified bands in the EEG from WS patients and controls. The third topographical map represents the result of channel-wise statistical inference, where t values from channels showing statistically significant differences were depicted and non-significant results were set at 0. A bar plot then followed, depicting the comparison of each channel. ***, *p *< 0.001, and non-significant results were not labeled.

When all channels are combined, EEG from WS patients exhibited significantly decreased power at delta (0.5117 ± 0.0196 compared to 0.5479 ± 0.0157, *p *< 0.001) and beta (0.065 ± 0.005 compared to 0.1328 ± 0.019, *p *< 0.001) bands, as well as significantly elevated theta (0.2145 ± 0.0071 compared to 0.1655 ± 0.0067, *p *< 0.001) and alpha (0.2516 ± 0.0136 compared to 0.1914 ± 0.0085, *p *< 0.001) bands, compared to controls (Figure 2). Regarding individual channels, the EEG from WS patients displayed significantly attenuated delta band power in the occipital region (O1), and all channels showed significantly higher power in theta band except in the prefrontal regions (Fp1, Fp2, and F4), the occipital region (O1 and O2) also exhibited significantly higher alpha band power, whereas no individual channel showed a significant difference at the beta band, likely due to higher variation in the control group (Figure 2).

**Figure 3:**
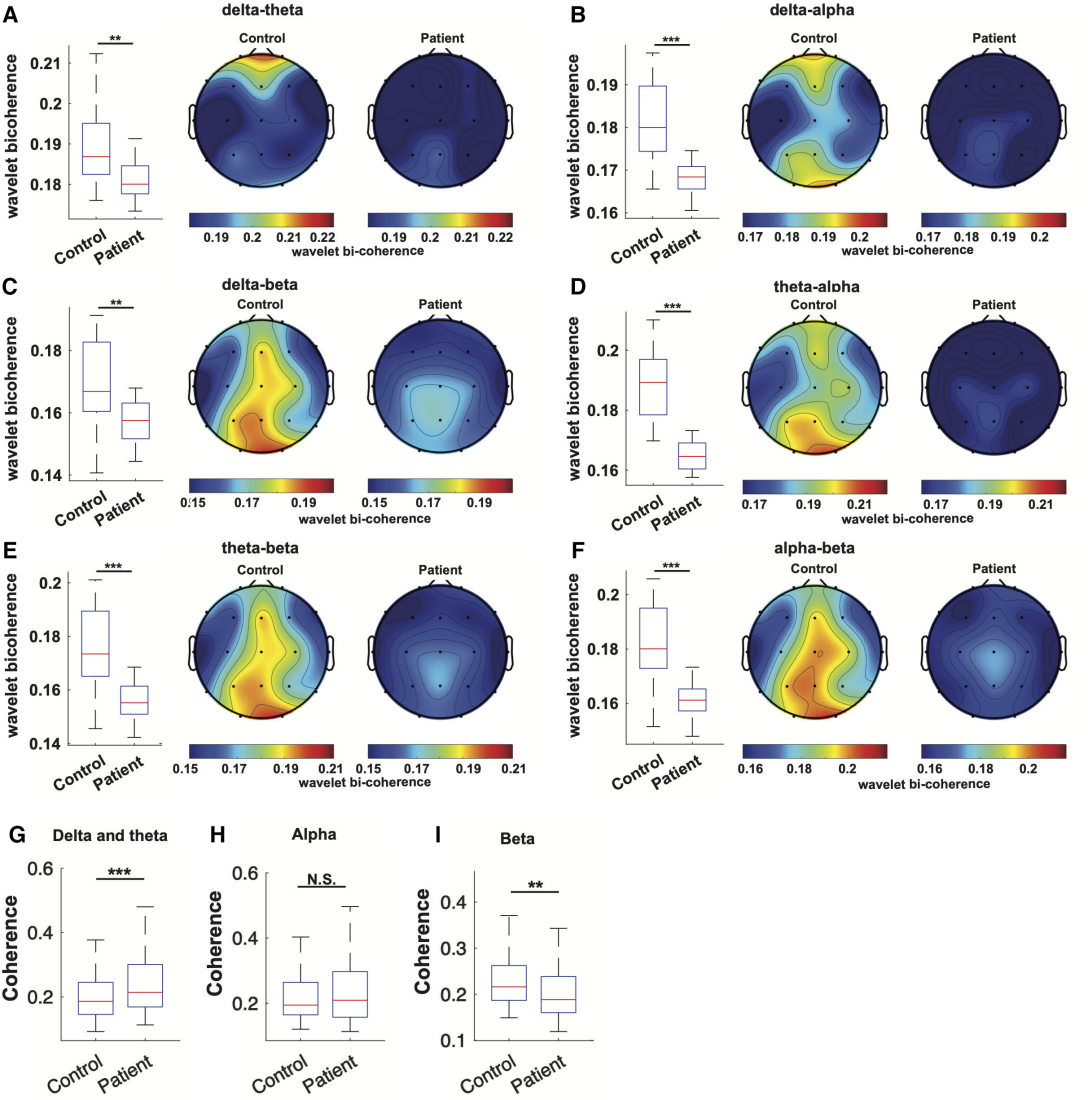
Cross-frequency coupling and bi-channel coherence of EEG from WS patients. (**A–F**) Wavelet bi-coherence between delta-theta band (**A**), delta-alpha band (**B**), delta-beta (**C**), theta-alpha band (**D**), theta-beta band (**E**), and alpha-beta band (**F**) in the EEG from WS patients and controls were compared respectively. For each frequency band pair, a comparison of overall bi-coherence was depicted as a boxplot, followed by topographical maps of channel-wise bi-coherence within the specified band pairs in the EEG from WS patients and controls. (**G–I**) Comparison of overall bi-channel coherence at the delta and theta band (**G**), alpha band (**H**), and beta band(**I**). The accompanying topographical illustration is available in Supplementary Figure S2. **, *p *< 0.01, ***, *p *< 0.001. N.S., not significant.

WS patients also exhibited significantly lower cross-frequency coupling in all assessed frequency band pairs (delta-theta, 0.1814 ± 0.0046 compared to 0.1901 ± 0.0064, *p *= 0.0015; delta-alpha,0.168 ± 0.0052 compared to 0,1813 ± 0.0078, *p *< 0.001; delta-beta, 0.1573 ± 0.0058 compared to 0.1688 ± 0.0084, *p *= 0.0027; theta-alpha, 0.1647 ± 0.0055 compared to 0.1886 ± 0.0083, *p *< 0.001; theta-beta, 0.1562 ± 0.0062 compared to 0.1744 ± 0.0086, *p *< 0.001; and alpha-beta, 0.1616 ± 0.0065 compared to 0.1813 ± 0.0086, *p *< 0.001) when all channels are combined (Figures 3A–F), although subsequent individual channel-wise comparisons yield no statistically significant difference after multi-comparison correction (data not shown).

The overall bi-channel coherence at the delta/theta band was significantly higher in WS patients compared to controls (0.2422 ± 0.0149 compared to 0.2024 ± 0.0134, *p *< 0.001, Figure 3G), with the difference between individual channel pairs Fp2-T5, O1-Pz, and F8-T5 remaining statistically significant after multi-comparison correction (refer to Supplementary Figure S2A). The overall bi-channel coherence at the alpha band did not show a significant difference between EEG from WS patients and controls (0.2384 ± 0.013 compared to 0.2227 ± 0.0143, *p *= 0.1156, Figure 3H). Nonetheless, a single pair of channels, F4–F8, exhibited significantly lower coherence in WS patients (refer to Supplementary Figure S2B). Additionally, the overall bi-channel coherence at the beta band was significantly lower in the EEG from WS patients (0.2105 ± 0.0151 compared to 0.2318 ± 0.195, *p *= 0.0046, Figure 3I), while no individual pair of channels showed a statistically significant difference (refer to Supplementary Figure S2C).

Taken together, linear EEG characterization revealed that WS patients showed an altered power spectrum, decreased cross-frequency coupling, and abnormal brain connectivity.

### 3.2 WS patients showed reduced permutation entropy

**Figure 4:**
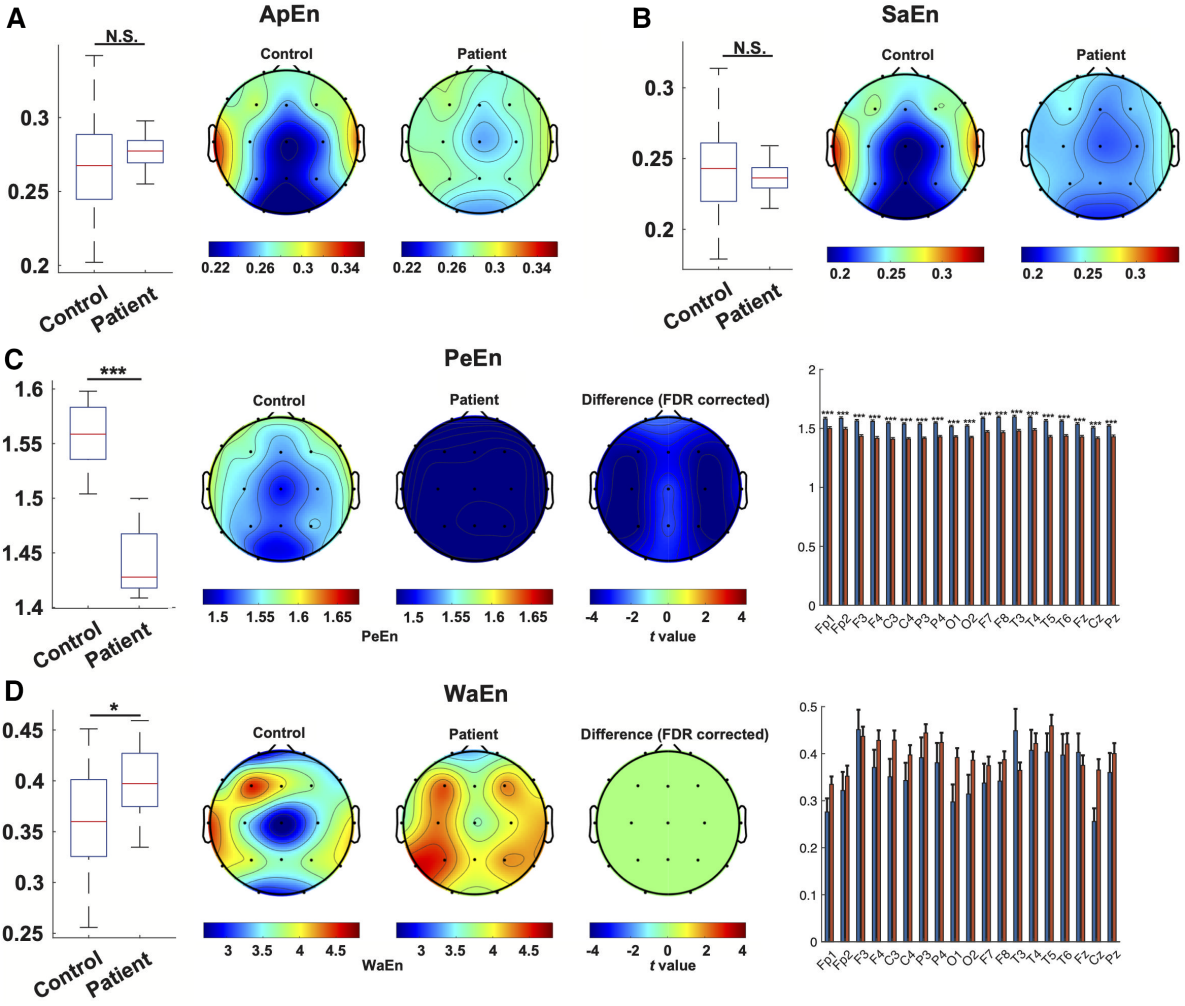
Nonlinear characterization of EEG from WS patients. The estimation of entropy with ApEn(**A**), SaEn (**B**), PeEn (**C**) and WaEn (**D**) in the EEG from WS patients and controls was compared. For each method, a comparison of overall entropy was depicted as a boxplot, followed by topographical maps of entropy in the EEG from WS patients and controls. In panels (**C,D**), the third topographical map shows the result of channel-wise statistical inference, where t values from channels showing a statistically significant difference were depicted, and non-significant results were set at 0. A bar plot then followed depicting the comparison of each channel. *, *p *< 0.05, ***, *p *< 0.001, N.S., not significant.

Assessing the nonlinear complexity of EEG signals using four entropy estimation methods between patients and controls, we found a significant and dramatic reduction in permutation entropy (PeEn) both globally combined (1.4411 ± 0.0133 compared to 1.5544 ± 0.0124, *p *< 0.001) and at each channel in the patient group. Additionally, wavelet entropy (WaEn) was slightly but significantly elevated in the EEG from WS patients (0.3995 ± 0.0205 compared to 0.3605 ± 0.0396, *p *= 0.011), although no individual channel showed a statistically significant difference. No difference was found between the two groups using approximate entropy (ApEn) and sample Entropy (SaEn) (Figure 4).

### 3.3 Association of structural-genetic etiologies with EEG features

**Figure 5:**
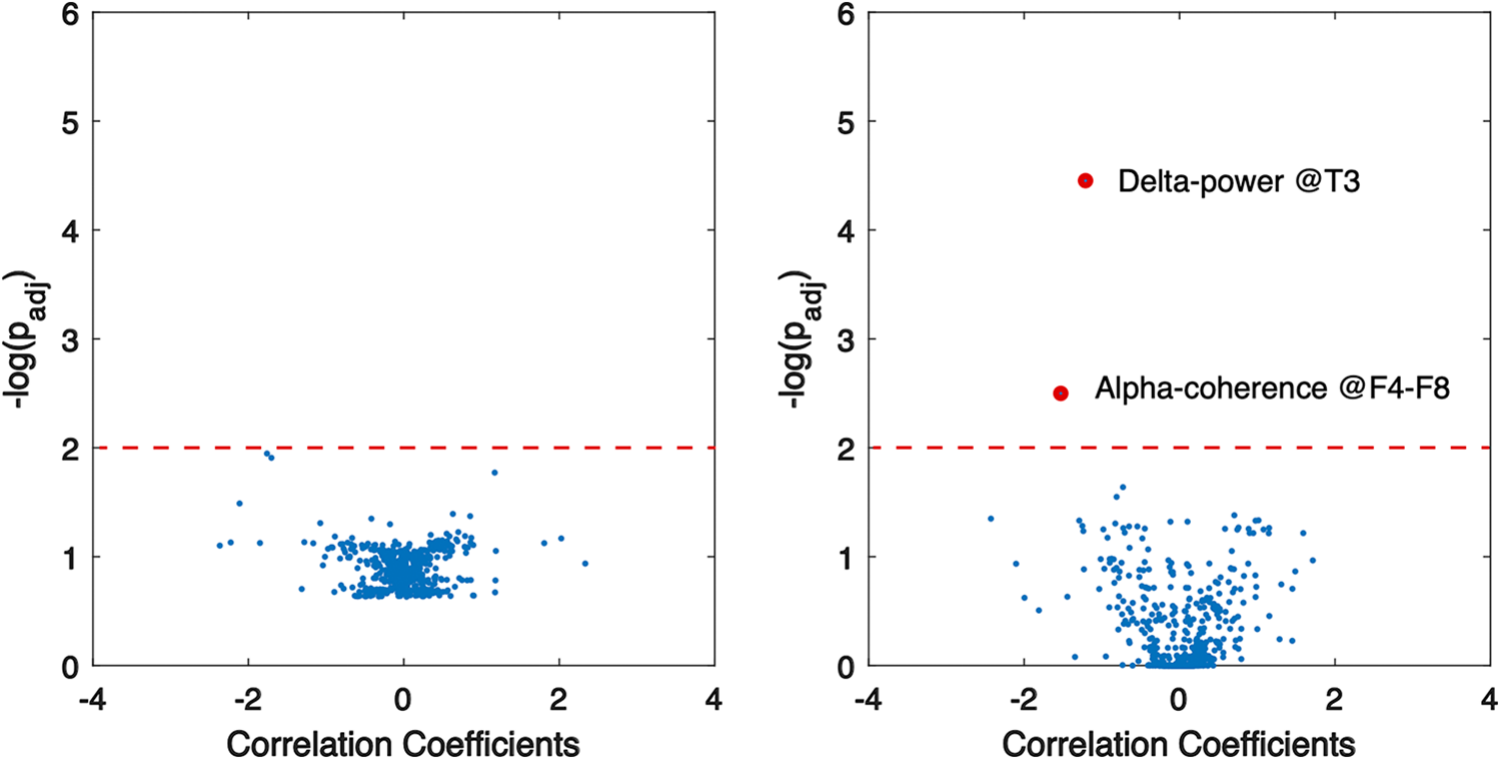
Association of EEG abnormalities with structural (left) and genetic (right) aberrancies in WS patients. Red dots indicate significant correlation, and dashed lines show the threshold of statistical significance.

Multivariate linear regression was employed to assess the correlation of structural and genetic etiologies of WS patients with EEG features. The results indicate that two factors—specifically, delta band power at the T3 region and coherence at the alpha band between F4–F8—showed a significant correlation with genetic abnormalities (Figure 5), whereas no significant association between structural factors and EEG features was revealed.

## 4 Discussion

In the present study, we observed a significant increase in global theta activity and a decrease in delta activity coupled with increased alpha activity in occipital regions in interictal EEG recordings of WS patients compared to controls. Additionally, beta-band activity was significantly lower in WS patients, although no specific region was identified in the subsequent channel-wise analysis. Delta activities are proposed to be associated with functional cortical inhibition (21), the attenuation of delta power in the occipital lobe could therefore reflect impaired inhibition specifically within the visual cortex. This disinhibition might also contribute to the excessive neuronal firing and the spread of seizure activity observed in WS. Our findings differ from previous reports. Burroughs et al. reported strengthened delta activity and attenuated theta, beta, and gamma activity in WS patients (15), while Smith et al. reported that WS patients showed global and broadband strengthening of the EEG power (10). The discrepancy between the present study and previous reports may be attributed to the exclusion of hypsarrhythmia activity in the present study, as hypsarrhythmia EEG patterns in WS patients have been associated with increased broadband amplitude and strengthened delta/alpha activities (11). Converging evidence suggests that theta activities are associated with cognitive conflict or errors (22), and the association begins as early as infancy (23). Dysregulation of theta activities in WS patients may reflect a continuous uncertain state, potentially leading to anxiety (24) and cognitive deficiency (25). High-gamma oscillations (40–150 Hz) have been associated with epileptogenicity and pathological brain lesions in WS patients (13, 26). Unfortunately, these were not assessed in the present study due to the limited sampling rate.

Cross-frequency coupling between neuronal oscillations plays a crucial role in neural functioning (27). High-frequency brain activities are associated with local processing, while low-frequency rhythms can travel across distant brain regions. Therefore, cross-frequency coupling facilitates the exchange of information between large-scale brain networks and local cortical processes, enabling functional integration across different spatiotemporal scales and effective computation (28). A previous study demonstrated that the coupling between interictal fast ripples (100–600 Hz) and the phase of slow wave activities (2–3 Hz) could differentiate treatment refractoriness in WS patients (14). In our present study, we observed an overall decrease in cross-frequency coupling across all assessed band pairs in WS patients, although no significant difference was identified at the single-channel level. This finding indicates that WS patients face significant challenges in coordinating global and local neural computations, which aligns with the neuro-psychomotor deficiencies commonly seen in WS patients.

Coherence between two brain regions indicates brain connectivity and reflects the functional integrity of the brain. Burroughs et al. reported that short-distance bi-channel coherence is decreased in frontal regions, whereas longer-distance bi-channel coherence is increased at broadband in the EEG of WS patients (15). The authors therefore proposed aberrant coherences may contribute to the developmental delay in WS. Moreover, Smith et al. recently reported that more than 80% of inter-channel connections at the delta band were increased in WS patients compared to controls (10). In our present study, we found that bi-channel coherence in EEG recordings of WS patients was significantly elevated at lower frequencies (i.e., delta-theta band), while it was decreased at higher frequencies (i.e., beta band). Despite methodological differences, increased inter-channel connectivity at the beta band in EEG recordings of WS patients was consistent among previous and present studies. This indicates that increased connectivity at the beta-band could serve as a biomarker for WS patients, whereas further investigations are warranted to elucidate the specific mechanism of connectivity alterations in WS and their functional and therapeutic implications.

Nonlinear characteristics are of particular significance in the investigation of intricate systems such as the human brain. Notably, entropy analysis of EEG signals has proven effective in the classification of various brain states, including distinguishing between awake and anesthetized states (29), as well as identifying healthy and epileptic conditions (30). A recent study by Chu et al. revealed a global reduction in EEG complexity, as assessed by multiscale entropy, in WS patients, which was found to be reversible with anti-epileptic drugs (16). Smith et al. observed that WS patients exhibited significantly higher Shannon entropy at the delta band, but decreased permutation entropy (PeEn) at both the delta and theta bands (10). In the present study, we investigated four entropy estimation methods—Approximate Entropy (ApEn), Sample Entropy (SaEn), Permutation Entropy (PeEn), and Wavelet Entropy (WaEn)—in interictal EEG recordings of WS patients. Interestingly, PeEn emerged as the most effective nonlinear feature in differentiating WS patients from controls, displaying minimal inter-subject variation and aligning with previous findings. While both ApEn and SaEn did not demonstrate significant differences, WaEn exhibited a slight increase in WS patients. PeEn assesses signal complexity by analyzing patterns of relative change between neighboring values, rather than absolute distances within an embedding space as with ApEn and SaEn (31), rendering it more robust when applied to noisy data such as EEG recordings (29). Although further research is necessary to validate these findings, the results suggest that permutation entropy holds significant promise as a nonlinear EEG biomarker for WS.

Variant patterns of hypsarrhythmia have been implicated in the etiology of WS (32). However, the correlation of computational EEG features with the etiology of WS has not been previously reported. One of the objectives of this study was to address this gap by examining the impact of genetic and structural aberrancy on abnormal linear and non-linear interictal EEG features in WS patients. Our findings revealed only 2 out of 969 features to be linked to genetic brain abnormalities in WS, and none were associated with causative structural variants. These results suggest that diverse etiological factors may give rise to converging pathology, leading to common symptomatic manifestations in individuals with WS.

The present study is subjected to several limitations. Firstly, the relatively small size of the patient and control cohorts may result in low statistical power. Secondly, the limited sampling rate prevented the analysis of EEG components with higher frequency. Thirdly, the present study only analyzed data from the pre-treatment state, thus further exploration is needed to understand how these findings may reflect drug response or prognosis.

In summary, interictal EEG patterns in WS patients revealed prominent global elevation of theta activity and decreased delta activity, coupled with increased alpha activity in occipital regions. Cross-frequency coupling was weaker in WS patients, and they also exhibited minor bi-channel correlation attenuation. Among the entropy estimation methods studies, PeEn, but not ApEn, SaEn, or WaEn, demonstrated a global reduction of complexity of interictal EEG of WS patients. While structural aberrancies were not found to contribute to alterations in interictal EEG in WS patients, genetic abnormalities were associated with subtle shifts in the power spectrum and inter-channel coherences. This study proposes that a combined global strengthening of theta activity and global reduction of permutation entropy could serve as a computational EEG biomarker of West syndrome.

## Data Availability

The original contributions presented in the study are included in the article/**Supplementary Material**, further inquiries can be directed to the corresponding author.
