# Supplementary figures and images for: Interictal EEG features as computational biomarkers of West syndrome
Source: Front Pediatr. 2024 Jun 5;12:1406772. doi: 10.3389/fped.2024.1406772 (PMC11188363; doi:10.3389/fped.2024.1406772)

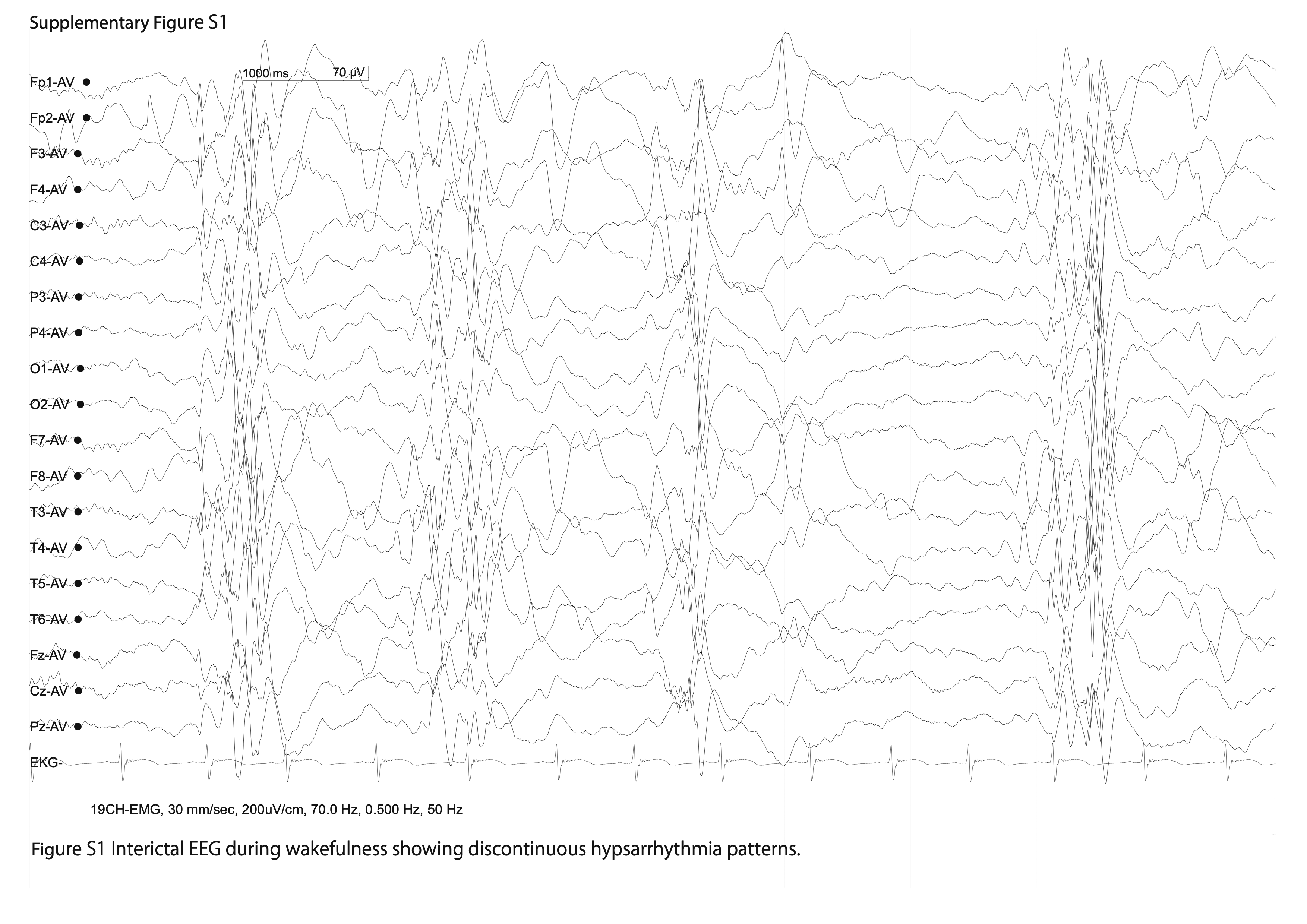

Supplement: Supplementary file 1 [file Image1.tiff]

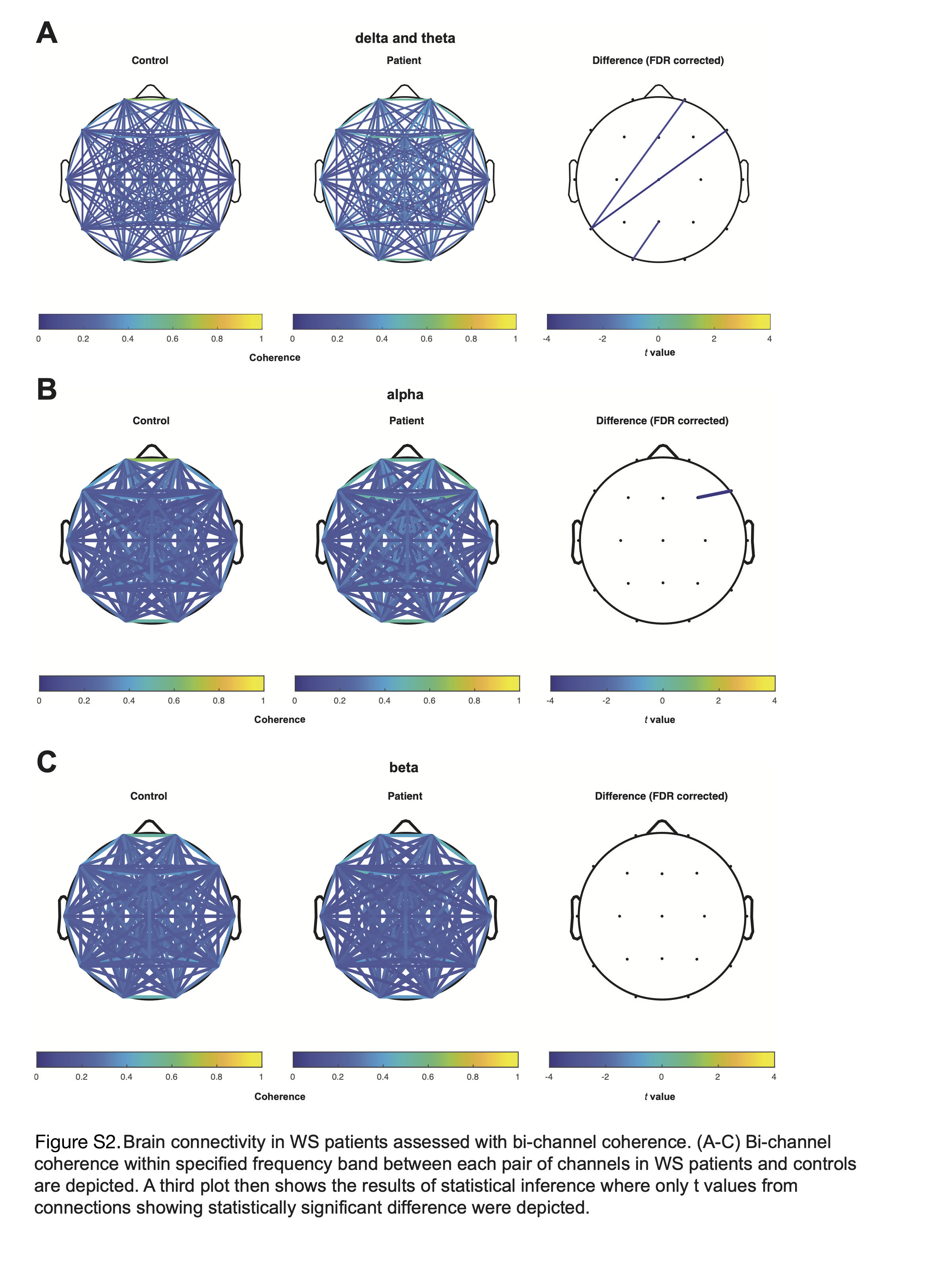

Supplement: Supplementary file 2 [file Image2.tif]
